# Vaccinia Virus Protein C6 Inhibits Type I IFN Signalling in the Nucleus and Binds to the Transactivation Domain of STAT2

**DOI:** 10.1371/journal.ppat.1005955

**Published:** 2016-12-01

**Authors:** Jennifer H. Stuart, Rebecca P. Sumner, Yongxu Lu, Joseph S. Snowden, Geoffrey L. Smith

**Affiliations:** Department of Pathology, University of Cambridge, Cambridge, United Kingdom; University of Texas Health Science Center at San Antonio, UNITED STATES

## Abstract

The type I interferon (IFN) response is a crucial innate immune signalling pathway required for defense against viral infection. Accordingly, the great majority of mammalian viruses possess means to inhibit this important host immune response. Here we show that vaccinia virus (VACV) strain Western Reserve protein C6, is a dual function protein that inhibits the cellular response to type I IFNs in addition to its published function as an inhibitor of IRF-3 activation, thereby restricting type I IFN production from infected cells. Ectopic expression of C6 inhibits the induction of interferon stimulated genes (ISGs) in response to IFNα treatment at both the mRNA and protein level. C6 inhibits the IFNα-induced Janus kinase/signal transducer and activator of transcription (JAK/STAT) signalling pathway at a late stage, downstream of STAT1 and STAT2 phosphorylation, nuclear translocation and binding of the interferon stimulated gene factor 3 (ISGF3) complex to the interferon stimulated response element (ISRE). Mechanistically, C6 associates with the transactivation domain of STAT2 and this might explain how C6 inhibits the type I IFN signalling very late in the pathway. During virus infection C6 reduces ISRE-dependent gene expression despite the presence of the viral protein phosphatase VH1 that dephosphorylates STAT1 and STAT2. The ability of a cytoplasmic replicating virus to dampen the immune response within the nucleus, and the ability of viral immunomodulators such as C6 to inhibit multiple stages of the innate immune response by distinct mechanisms, emphasizes the intricacies of host-pathogen interactions and viral immune evasion.

## Author Summary

In response to a viral infection, infected host cells mount an early, innate immune response to limit viral replication and spread. Type I interferons (IFNs) are produced by a cell when a viral infection is detected and are a crucial aspect of this early immune response. IFNs are released from the infected cell and can act on the infected cell itself or neighbouring cells to initiate a signalling pathway that results in the production of hundreds of anti-viral proteins. In this work we investigated a vaccinia virus protein called C6, a known inhibitor of type I IFN production. Here we show that C6 also inhibits signalling initiated in response to type I IFNs, therefore providing a dual defence against this essential immune response. The results show that, unlike the majority of viral inhibitors of IFN signalling, C6 inhibits the signalling pathway at a late stage once the proteins required for IFN-stimulated gene transcription have reached the nucleus and bound to the DNA. This work illustrates the complex relationship between infecting viruses and the host immune response and further investigation of the mechanism by which C6 inhibits this important immune pathway will likely increase our knowledge of the pathway itself.

## Introduction

The innate immune response is the first line of defense against invading pathogens. Interferons (IFNs) are one of the key players in this early response to infection and are particularly important to protect against viruses, as can be seen by the increased susceptibility of IFNα/β receptor (IFNAR) knock out mice to viral infections [1]. There are two main branches to the IFN response; their production and the signalling initiated in response to the binding of secreted IFNs to their receptors at the cell surface.

Type I IFNs, which include IFNβ, several IFNα variants and other tissue or species-specific members, are produced directly in response to virus detection by cellular pattern recognition receptors (PRRs). Upon recognition of pathogen associated molecular patterns (PAMPs) such as viral DNA or RNA, PRRs activate several signalling pathways many of which converge on the kinases TANK-binding kinase (TBK1) and IκB kinase-ε (IKKε). These kinases, in complex with adaptor proteins such as TANK, NAK-associated protein 1 (NAP1) or similar to NAP1 TBK1 adaptor (SINTBAD), phosphorylate the transcription factor IFN regulatory factor 3 (IRF-3). Once phosphorylated, IRF-3 dimerises and translocates into the nucleus and, in combination with other transcription factors, drives transcription from promoters containing cognate binding sites, including the IFNβ promoter [2].

Once produced and secreted from cells, type I IFNs can act in a paracrine or autocrine fashion by binding to the IFNAR, which is composed of the two subunits IFNAR1 and IFNAR2. The binding of type I IFN to the receptor complex leads to the cross activation of the two Janus protein kinases, Tyk2 and Jak1 that are bound to the cytoplasmic domains of the IFNAR1 and IFNAR2, respectively. Once activated these kinases phosphorylate the transcription factors signal transducer and activator of transcription 1 (STAT1) and STAT2. These phosphorylated proteins then heterodimerise and bind to IRF-9 to form the IFN stimulated gene factor 3 (ISGF3) transcriptional activator complex. This tripartite complex translocates into the nucleus where it binds to IFN stimulated response elements (ISREs) found in the promoter of IFN stimulated genes (ISGs) and induces their transcription. The type I IFN signalling pathway and its regulation is reviewed in [3].

The importance of the IFN response for protection against viral infections is illustrated by the array of mechanisms and proteins used by viruses to evade and inhibit these cellular pathways, reviewed in [4]. Vaccinia virus (VACV) is a well-studied member of the *Poxviridae* and was the vaccine used in the eradication of smallpox [5]. It is a large DNA virus, with approximately 200 genes, that replicates exclusively in the cytoplasm of infected cells [6]. Between one third and one half of these 200 genes have been shown to have immunomodulatory or immunoevasive roles [7,8].

Many of these immunomodulatory proteins are able to inhibit type I IFN production, either through inhibition of the NF-κB pathway, for example VACV proteins B14 [9] and A49 [10], or through inhibition of the IRF-3 signalling pathway, as with VACV proteins A46 [11] and K7 [12]. In contrast, very few inhibitors have been identified that act post-IFN production. To date two VACV proteins are known to inhibit IFN signalling after type I IFN has been secreted from cells. B18 is a secreted VACV protein that binds type I IFN in solution and on the surface of cells and prevents its interaction with the IFNAR [13–15] and VH1 is a virally-encoded phosphatase that is packaged within virions [16] and dephosphorylates both STAT1 and STAT2, therefore acting as an intracellular inhibitor of JAK/STAT signalling [17,18].

C6 is a predicted member of the VACV Bcl2-like protein family, a family of 10 proteins whose previously studied members have various innate immune inhibitory functions [19–25]. It is expressed early during infection and its deletion attenuates the virus in both intranasal and intradermal models of infection in the mouse [26]. Despite being attenuated, VACV strains engineered to lack C6 showed enhanced immunogenicity in vivo [27,28]. Previously, C6 was shown to bind to the TBK1/IKKε adaptor proteins, SINTBAD, NAP1 and TANK, and prevents the TBK1/IKKε-dependent activation of IRF-3 and therefore inhibits the induction of type I IFNs [26]. Given many VACV proteins have been shown to have multiple functions, for example N1 that inhibits both NF-κB signalling [29] and apoptosis [23,30], and the observation that the hitherto only known function of C6 occurs in the cytoplasm of infected cells despite C6 being present in the nucleus and cytoplasm, we investigated whether C6 may have additional immunomodulatory functions.

In this study, VACV protein C6 is shown to be a dual function protein that inhibits type I IFN signalling as well as type I IFN production. Data presented show that the inhibition of IFNα-induced JAK/STAT signalling occurs at a late stage in the pathway, downstream of STAT phosphorylation, heterodimerisation, and nuclear translocation and downstream of ISGF3 binding to the ISRE, thus indicating an inhibitory function of C6 at or after formation of the transcriptional complex. Furthermore, C6 is shown to associate with the transcriptional activating domain (TAD) of STAT2, providing a plausible mechanism by which this viral protein could disrupt transcriptional complex formation. Interestingly, C6 was able to inhibit the transcriptional induction of all but one of the IFNα-dependent genes tested. This indicates that the step(s) inhibited by C6 downstream of ISGF3-ISRE interaction is likely to be conserved for a large number of ISGs, rather than a gene-specific transcriptional requirement. To our knowledge, this additional function of C6 makes C6 the first nuclear inhibitor of the IFN response encoded by the cytoplasmic-replicating DNA virus, VACV. The ability of VACV, a virus whose replication cycle takes place entirely within the cytoplasm of infected cells, to extend its influence into the host cell nucleus to inhibit crucial innate immune signalling pathways, highlights the complexity of virus-host interactions.

## Results

### C6 inhibits type I IFN signalling

**Fig 1:**
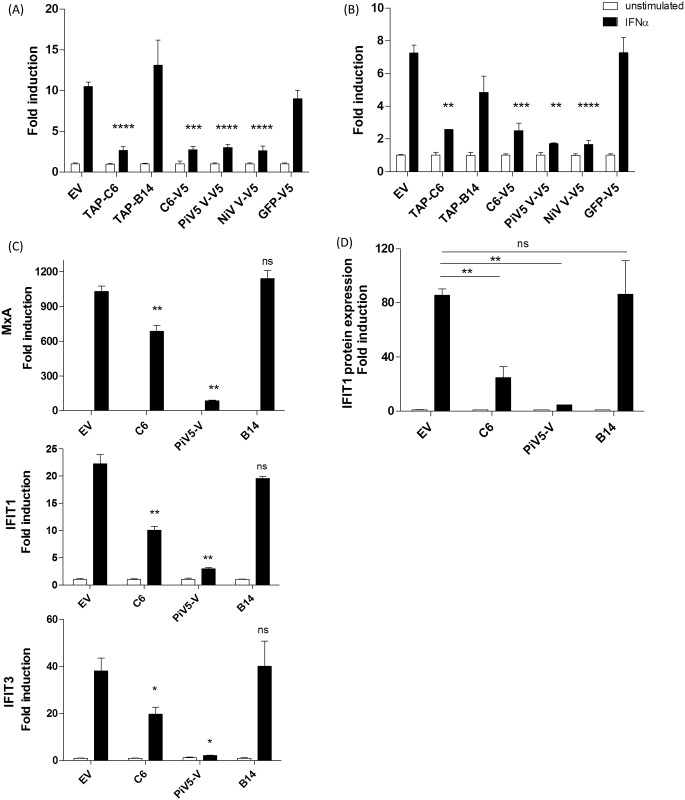
VACV protein C6 inhibits IFNα induced ISG expression. HEK-293T (**A**) and HeLa (**B**) cells were co-transfected in triplicate with an ISRE-luciferase reporter, a plasmid expressing renilla luciferase constitutively and expression vectors for the proteins shown. Twenty four hours post transfection cells were stimulated with 25 U/ml IFNα for 8 h. Firefly luciferase activity was measured by luminesence and normalised to a renilla luciferase expression as a control for transfection efficiency. (**C** and **D**) HeLa cells stably expressing the proteins shown were stimulated with 500 U/ml IFNα for 8 h. (**C**) mRNA was harvested from cells and used for qPCR analysis of ISG induction. (**D**) Cells were fixed, permeabilised, stained for IFIT1 protein expression and analysed by flow cytometry. Data are presented as the fold induction relative to the unstimulated control of each sample -/+ standard deviation. Statistics are relative to EV-stimulated control. *p<0.05 **p<0.01 ***p<0.001 ****p<0.0001.

Previously, C6 was characterised as an inhibitor of IFNβ production through inhibition of the IRF-3/7 signalling pathway [26]. However, many viral proteins are known to have multiple functions. To determine whether C6 was able to also inhibit signalling downstream of type I IFNs, its effect on the expression of an ISRE-dependent luciferase reporter gene (ISRE-luciferase) was assessed. HeLa and HEK293T cells were co-transfected with expression plasmids for ISRE-luciferase and V5- or TAP- tagged C6 or control proteins (S1 Fig). Cells were then stimulated with IFNα for 8 h and the expression of luciferase was measured by luminescence. Treatment of HEK293T and HeLa cells with IFNα led to an induction of luciferase expression and in both cell types this induction was significantly inhibited by the co-expression of either TAP-C6 (p<0.0001 and p<0.01 respectively) or C6-V5 (p<0.001 and p<0.001 respectively, Fig 1A and 1B). As expected the positive controls Nipah Virus V protein (NiV-V) and Parainfluenza virus 5 V protein (PiV5-V) that are known to inhibit IFN signalling [31,32] also inhibited IFNα-induced luciferase expression, whilst expression of GFP or VACV protein B14 had no inhibitory effect.

To confirm the ability of C6 to inhibit type I IFN signalling, the effect of C6 on the induced transcription of endogenous ISGs was assessed next. HeLa cells stably expressing GFP alone (EV) or GFP in combination with V5 tagged- C6, PiV5-V or B14 were stimulated with IFNα for 8 h. RNA was extracted from these cells and used for qPCR analysis of ISG mRNA expression. IFNα treatment of cells resulted in induction of the well-characterised ISGs tested, including interferon-induced protein with tetratricopeptide repeats 1 (IFIT1), IFIT3 and MxA. The presence of C6 significantly inhibited the induction of gene expression when compared to both the EV and B14 controls (Fig 1C). Once again PiV5-V expression also inhibited the induction of ISG expression as expected (Fig 1C).

Finally, these stably transduced cells were used to confirm the ability of C6 to inhibit IFNα-induced gene expression at the protein level using flow cytometry analysis of IFIT1 expression. Cells were stimulated with IFNα for 8 h and then fixed, permeabilised and stained with an anti-IFIT1 antibody. Stained cells were analysed by flow cytometry to assess for IFIT1 protein expression. Once again treatment of EV transduced cells with IFNα resulted in an induction of IFIT1 expression, which was significantly inhibited by expression of C6 or the positive control, PiV5-V (p<0.01, Fig 1D). In contrast, expression of B14 had no effect on IFIT1 expression. Together these data show that C6 inhibits IFNα mediated gene expression at both the mRNA and protein level.

### The ability of C6 to inhibit signalling downstream of IFNα treatment, is not a result of its ability to inhibit TBK1/IKKε function

The IRF-3 signalling pathway is initiated in response to detection of viral RNA, DNA or proteins by PRRs in the cell and leads to the activation of the kinases TBK1 and IKKε. These kinases then phosphorylate IRF-3 causing its translocation into the nucleus from where it drives transcriptional activation of a number of target genes including IFNβ. C6 inhibits IRF-3 signalling at the level of TBK1 and IKKε, preventing the nuclear translocation of IRF-3 [26]. Several studies have described potential crosstalk between the IRF-3 and JAK/STAT signalling pathways, which together constitute the type I IFN response. In addition, IRF-3 is known to cause the transcriptional activation of a subset of ISRE containing gene promoters directly [33] and an additional phosphorylation event on STAT1 by IKKε is required for the full induction of approximately 30% of IFNα-responsive ISGs [34].

To rule out that the inhibitory effect of C6 on type I IFN signalling was an indirect consequence of its ability to interfere with TBK1 and IKKε function, and that this inhibitory activity was instead a novel function for C6, the effect of a TBK1/IKKε specific inhibitor, BX795, on the ISRE-dependent reporter gene assay was assessed. Cells transfected with the ISRE-luciferase reporter were treated for 3 h with BX795 before stimulation with IFNα for a further 6 h in the continued presence of BX795. BX795 treatment had no inhibitory effect on the IFNα-dependent induction of firefly luciferase in the cells tested, when compared to a carrier (dimethyl sulphoxide, DMSO)-treated control (S2 Fig). To confirm the effectiveness and specificity of BX795 in this assay, BX795 treatment was also used in conjunction with firefly luciferase under the control of promoters responsive to the IRF-3 and NF-κB signalling pathways. Cells transfected with these luciferase reporters were stimulated with polyI:C and IL-1β respectively. Whilst treatment with BX795 had no effect on IL-1β-induced activation of the NFκB reporter gene, the induction of the IRF-3-responsive promoter by poly I:C was significantly reduced (p<0.001), indicating that the inhibitor was effective at the doses used (S2 Fig). These data indicate that inhibition of TBK1 and IKKε does not cause the inhibition of ISRE-dependent gene expression that is seen in the presence of C6, implying that this is instead a novel function of C6.

### C6 does not inhibit the IFNα-induced phosphorylation or dimerisation of STAT1 and STAT2

**Fig 2:**
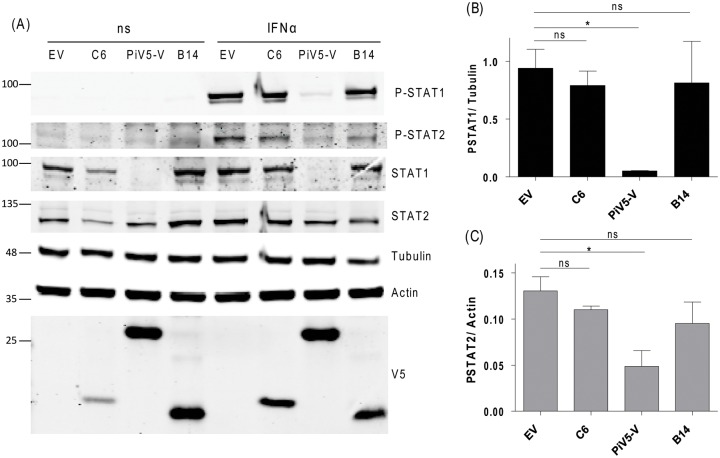
C6 does not inhibit the IFNα-induced phosphorylation of STAT1 or STAT2. HeLa cells stably expressing the proteins shown were stimulated with IFNα (1000 U/ml for 1 h). Cells were harvested and cell lysates subjected to SDS-PAGE and immunoblotting to assess levels of STAT1 and STAT2 phosphorylation (**A**). Samples were also immunoblotted for alpha tubulin and actin as controls. Positions of molecular mass markers are shown on the left of the figure. Quantification of triplicate samples of phosphorylated STAT1 (**B**) and phosphorylated STAT2 (**C**) proteins was performed using Odyssey software (LICOR) and are shown relative to a constant house-keeping gene. P<0.05. Immunoblots were performed at least twice and a representative figure is shown.

To determine how C6 inhibits the cellular response to IFNα, the phosphorylation status of STAT1 and STAT2 in cells expressing C6 following IFNα treatment was examined. HeLa cells stably expressing C6 or control proteins were stimulated with IFNα for 45 mins. Cell lysates were used for immunoblot analysis of levels of phosphorylated STAT1 and STAT2, including quantification of proteins using Odyssey software (LICOR). Neither C6 nor the negative control protein B14 inhibited the IFNα-induced phosphorylation of STAT1 or STAT2, whereas PiV5-V protein inhibited the phosphorylation of both proteins as expected (Fig 2).

**Fig 3:**
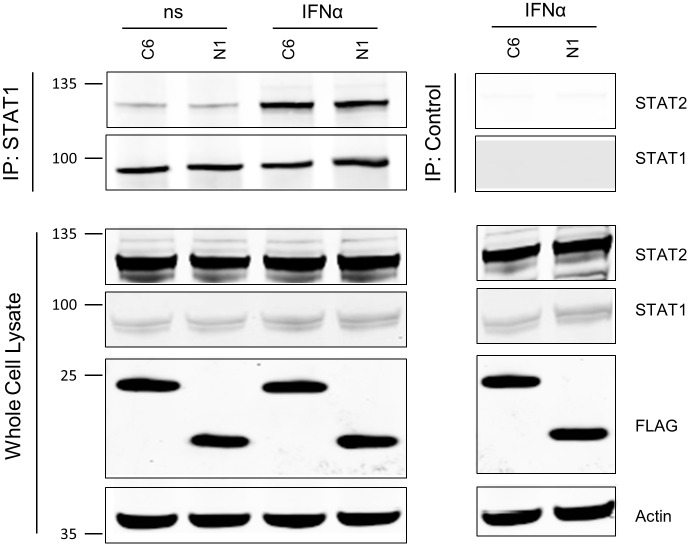
C6 does not inhibit the IFNα-induced interaction between STAT1 and STAT2. HEK293T cells were transfected with the TAP-tagged C6 or N1 plasmids. Sixteen hours post transfection cells were stimulated with IFNα (500 U/ml for 45 min). Cells were lysed and an immunoprecipitation against STAT1 was performed on cell lysates. Immunoblot analysis was used to determine the amount of STAT2 bound to STAT1 in each condition. Immunoblots were performed at least twice and a representative figure is shown. Positions of molecular mass markers are shown to the left of the immunoblots.

Following phosphorylation, STAT1 and STAT2 heterodimerise and bind to IRF9 to form the ISGF3 complex. This complex then translocates into the nucleus where it drives expression of genes with an ISRE in their promoter. To determine whether C6 prevents the dimerisation of STAT1 and STAT2, an immunoprecipitation of STAT1 from IFNα-stimulated cells was performed in cells transiently transfected with C6 or N1, a VACV Bcl-2-like protein that does not inhibit IFNα signalling. Immunoblot analysis showed that following IFNα stimulation an increased amount of STAT2 was found associated with STAT1. However, C6 did not alter the amount of STAT2 bound to STAT1 either before or after stimulation (Fig 3).

### C6 inhibits IFNα signalling at a step downstream of ISGF3 complex nuclear translocation

**Fig 4:**
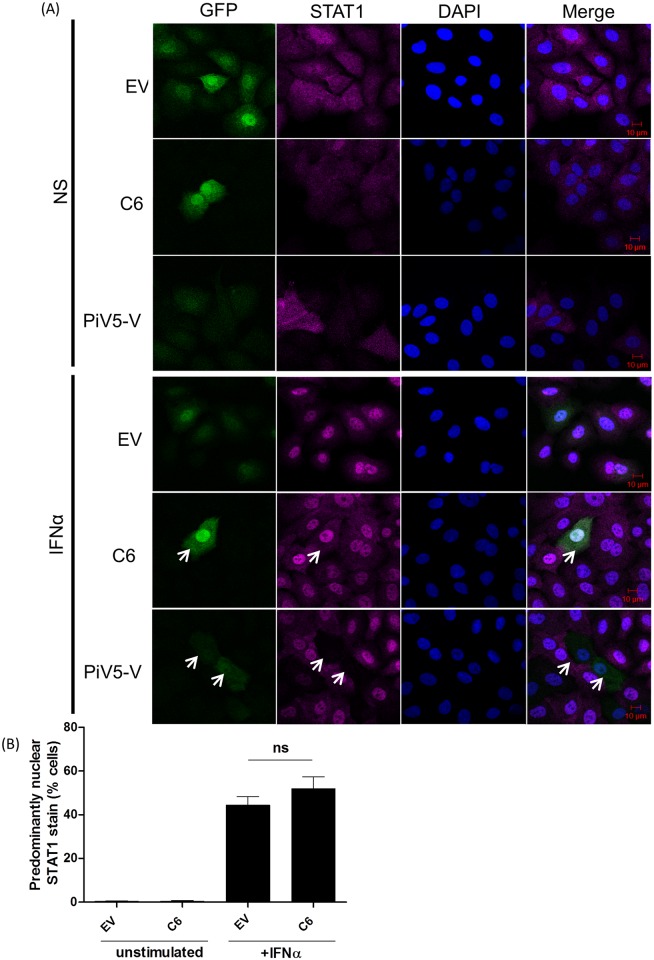
C6 does not inhibit the IFNα-induced nuclear translocation of STAT1. (**A**) HeLa cells stably expressing the stated proteins were stimulated with IFNα (1000 U/ml) for 1 h. Cells were then fixed, permeabilised and stained for STAT1. Protein localisation was determined by confocal microscopy. Three slides of each condition were made and one hundred GFP-positive cells on each slide were examined. The percentage of cells with a nuclear STAT1 (**B**) protein stain is shown.

**Fig 5:**
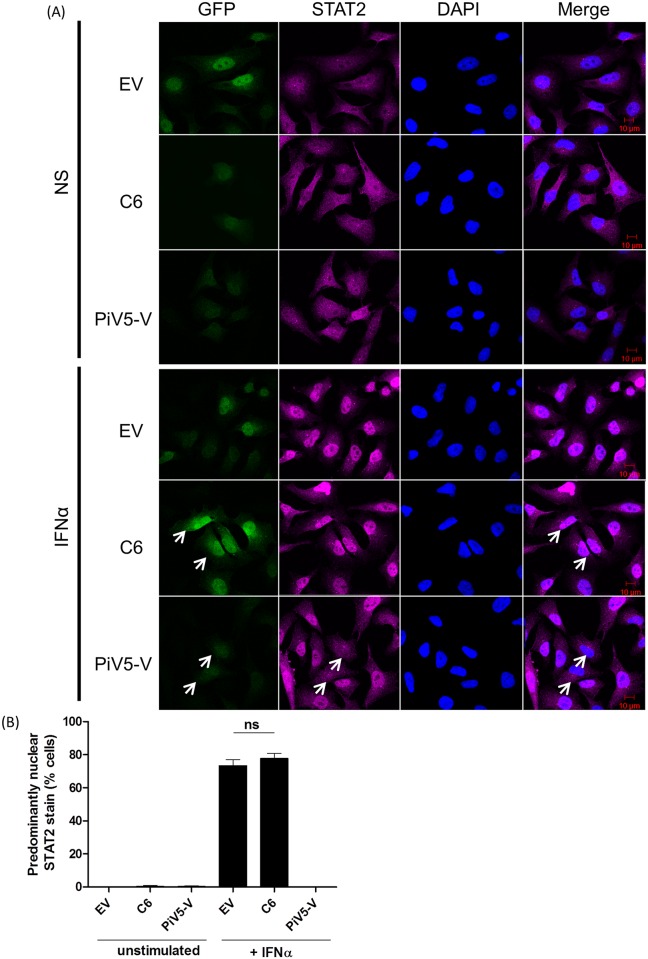
C6 does not inhibit the IFNα-induced nuclear translocation of STAT2. (**A**) HeLa cells stably expressing the stated proteins were stimulated with IFNα (1000 U/ml) for 1 h. Cells were then fixed, permeabilised and stained for STAT2. Protein localisation was determined by confocal microscopy. Three slides of each condition were made and one hundred GFP-positive cells on each slide were examined. The percentage of cells with a nuclear STAT2 (**B**) protein stain is shown.

Following its formation, the ISGF3 complex translocates into the nucleus. To assess whether this nuclear translocation was inhibited by C6 the localization of STAT1 and STAT2 before and after IFNα stimulation was assessed by confocal microscopy. HeLa cells stably expressing GFP alone (EV) or in combination with V5-tagged C6 or PiV5-V were stimulated with 500 U/ml IFNα for 1 h. Cells were then fixed, permeabilised and stained for endogenous STAT1 (Fig 4A) or STAT2 (Fig 5A). Following IFNα stimulation the percentage of cells showing a nuclear stain for STAT1 and STAT2 increased to approximately 50% (Fig 4B) and 70% (Fig 5B), respectively. These localization patterns were not altered by C6 or the negative control, EV. The localisation of STAT1 could not be assessed in PiV5-V expressing cells due to its degradation by this viral protein, however, PiV5-V expression inhibited the translocation of STAT2 completely (Fig 5A). Together these data indicate that C6 does not inhibit the pathway prior to ISGF3 complex formation and nuclear translocation.

**Fig 6:**
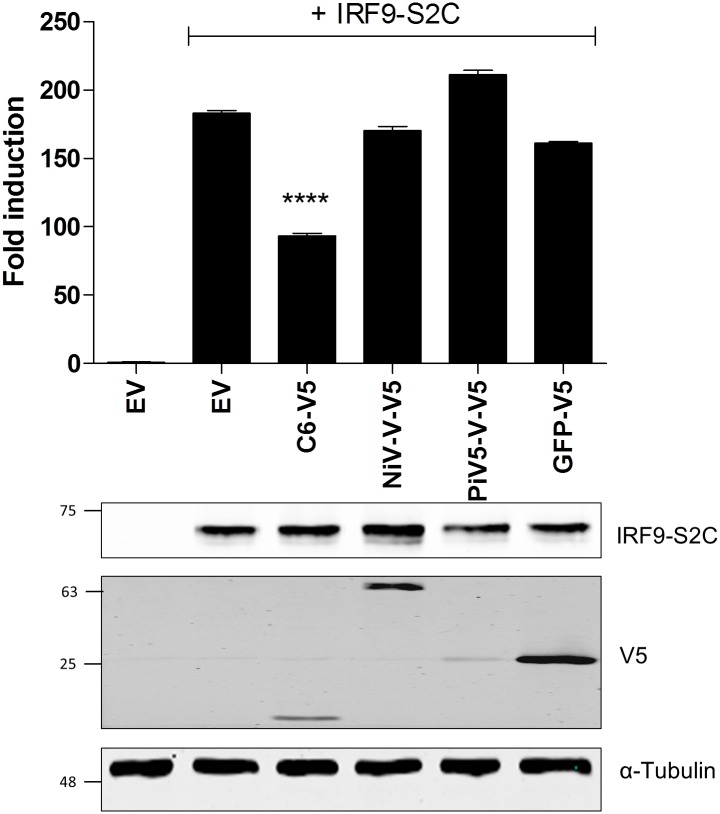
C6 can inhibit induction of ISRE-luciferase by a constitutively active ISGF3 mimic, IRF9-S2C. HEK293T cells were transfected with plasmids encoding firefly luciferase under the control of an ISRE promoter, a constitutively expressed renilla luciferase and the other proteins shown. Cells were harvested after 24 h and the expression of firefly luciferase was measured and normalised to renilla luciferase expression. Results show the fold induction of firefly luciferase relative to the EV only control. Immunoblot analysis shows expression of IRF-9-S2C and the V5-tagged proteins in each sample. Immunoblots were performed at least twice and a representative figure is shown. Positions of molecular mass markers are shown to the left of the immunoblots. ****p<0.0001.

To obtain additional evidence that the inhibitory effect of C6 on IFNα signalling is downstream of ISGF3 complex formation, a plasmid encoding IRF-9 fused to the C-terminal region (amino acids 747–851) of the transcriptional activation domain of STAT2 (referred to as IRF9-S2C) was utilised. Previously, this fusion protein, which overcomes the need for ISGF3 complex formation, has been shown to act as a constitutively active ISGF3-like transcriptional activator in the absence of IFN stimulation [35]. When a plasmid encoding IRF9-S2C was co-transfected into HEK293T cells along with the ISRE-luciferase reporter gene, a large increase in firefly luciferase expression was observed in cells expressing IRF9-S2C relative to those transfected with empty vector (EV) only (Fig 6, columns 1 and 2). Interestingly, when co-transfected into cells, C6 inhibited IRF9-S2C-driven ISRE reporter activity significantly (p<0.0001), whereas, neither PiV5-V protein nor NiV-V protein showed any inhibitory activity (Fig 6). This is in keeping with the known ability of these two viral proteins to inhibit the IFNα signalling pathway upstream of ISGF3 complex formation [31,32]. These data confirm that C6 inhibits IFNα signalling at a late stage following ISGF3 complex formation.

### C6 does not prevent the binding of IRF9-S2C to the ISRE

**Fig 7:**
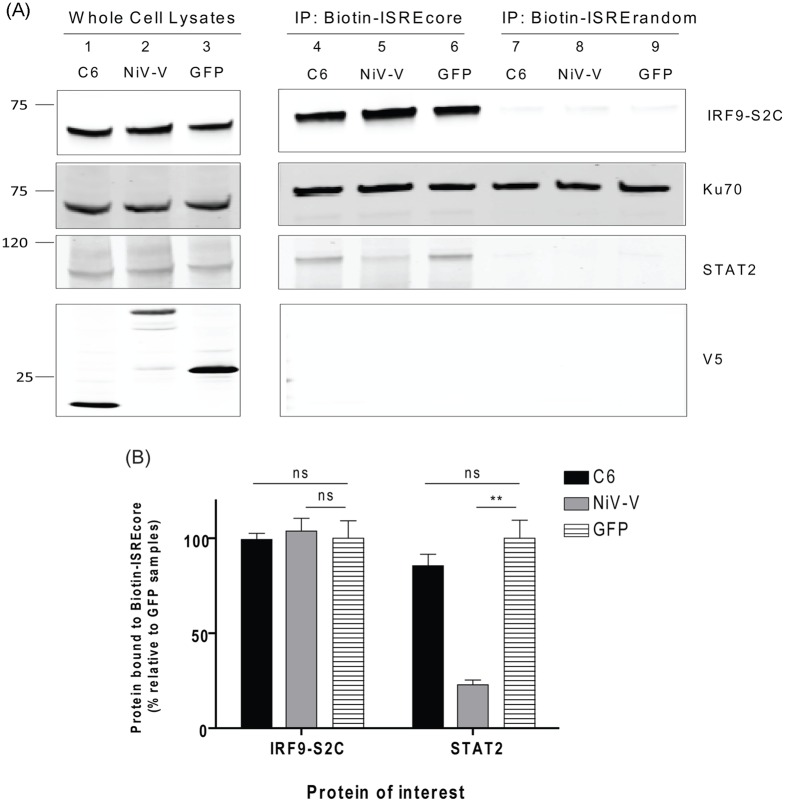
C6 does not prevent the binding of IRF9-S2C or endogenous STAT2 to the ISRE. HEK293T cells were transfected with plasmids expressing IRF9-S2C (lanes 1–9) and either V5-tagged C6 (lanes 1, 4 and 7), NiV-V (lanes 2, 5 and 8) or GFP (lanes 3, 6 and 9). Sixteen hours post transfection cells were harvested and lysates were incubated with polyd(I:C) for 30 min. Biotin-labelled ISRE probes (ISREcore lanes 4–6, and ISRErandom lanes 7–9) were then added for 1.5 h before addition of streptavidin agarose beads for a further 3.5 h. Beads were washed four times and proteins were eluted and subjected to SDS-PAGE and immunoblotting (**A**). Band intensities were quantified and normalised to Ku70 immunoprecipitation (**B**). Immunoblots were performed at least twice and a representative figure is shown. Positions of molecular mass markers are shown to the left of the immunoblots. **p<0.01.

To establish whether C6 inhibited IFNα signalling by preventing the binding of the ISGF3 complex to the ISRE in promoters of ISGs, the ability of the IRF9-S2C fusion protein to bind the ISRE was assessed. HEK293T cells were transfected with plasmids expressing IRF9-S2C and V5-tagged C6 or control proteins and cell lysates were harvested 16 h later. A biotin-labelled ISRE probe optimised previously for ISGF3 binding (ISREcore) [36], or a control biotin-labelled ISRE sequence that was shown to lack ISGF3 binding (ISRErandom) [36] were incubated with cell lysates and streptavidin beads were used to immunoprecipitate the biotinylated DNA probe and associated proteins. C6 or GFP expression did not inhibit the binding of either IRF9-S2C or endogenous STAT2 to the biotin-labelled ISRE (Fig 7). In contrast, NiV-V protein was able to inhibit endogenous STAT2 binding to the ISRE but not binding of the IRF9-S2C construct as expected (Fig 7). Neither IRF9-S2C nor endogenous STAT2 bound to the ISRErandom control sequence as expected. Ku70, a known DNA binding protein, was used here as a control for DNA input and gel loading and was found to bind to both the ISREcore and ISRErandom DNA probes.

### C6 associates with the transactivation domain of STAT2

**Fig 8:**
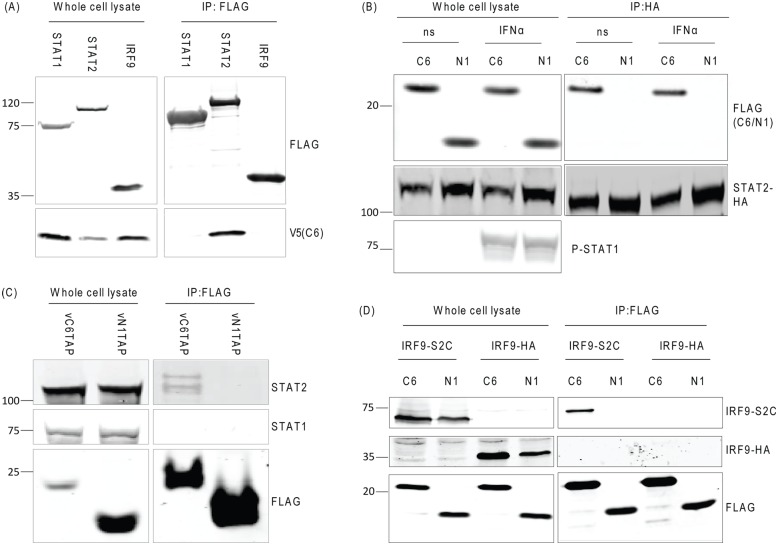
C6 interacts with the transactivation domain of STAT2. HEK293T cells were co-transfected with V5-C6 and TAP-tagged STAT1, STAT2 or IRF-9 expressing plasmids for 16 h (**A**), or co-transfected with TAP-tagged C6 or N1 and HA-STAT2 (**B**) or infected with the viruses shown for 16 h at 2 PFU/cell (**C**) or co-transfected with TAP-tagged C6 or N1 and IRF9-S2C or HA-IRF9 expressing plasmids for 16 h (**D**). In panel B, 16 h after transfection cells were stimulated with 100 units/ml IFNα for 1 h. (**A-D**) In all panels, cells were then lysed and immunoprecipitations against the FLAG- epitope (**A**, **C** and **D**) and the HA- epitope (**B**) were performed. Proteins were eluted and subjected to SDS-PAGE and immunoblotting with the stated antibodies. Immunoblots were performed at least twice and a representative figure is shown. Positions of molecular mass markers are shown to the left of the immunoblots.

To investigate whether C6 interacts with any of the components of the ISGF3 complex, an immunoprecipitation assay using FLAG-tagged STAT1, STAT2 and IRF-9 was performed. Plasmids expressing these proteins were co-transfected into HEK293T cells along with a V5-tagged C6 expression vector. Immunoprecipitation with anti-FLAG beads co-precipitated C6 with STAT2 but not with STAT1 or IRF-9 (Fig 8A).

To determine if the interaction between C6 and STAT2 was affected by IFN stimulation, HeLa cells were transfected with plasmids expressing HA-tagged STAT2 and either TAP-tagged C6 or N1 then mock-stimulated or stimulated with IFNα prior to immunoprecipitation (Fig 8B). This showed that the interaction between C6 and STAT2 did not require prior stimulation with IFN.

To confirm the interaction between C6 and STAT2 and to ascertain whether it occurs at endogenous protein levels and during viral infection, HEK293T cells were infected with VACVs expressing TAP-tagged C6 or N1 under the natural promoters for these genes. Cells were then lysed and immunoprecipitations were performed against the FLAG epitope in the TAP tag of these viral proteins. Once again C6 associated with STAT2 and not STAT1, whilst N1 did not associate with either protein (Fig 8C), confirming the specific interaction between STAT2 and C6 at endogenous protein levels during viral infection.

As C6 inhibits IFNα signalling initiated by IRF9-S2C expression, its ability to interact with this fusion protein, which contains only the C-terminal 104 aa of the STAT2 transactivation domain, was assessed. To this end, HEK293T cells were co-transfected with either IRF9-S2C or HA-IRF-9 and C6-TAP or N1-TAP. Immunoprecipitation with anti-FLAG beads showed association between IRF9-S2C and C6 but not between C6 and HA-IRF-9 (Fig 8D). This indicates that C6 associates with the final 104 aa of the STAT2 transactivation domain, a region known to be important for recruitment of downstream chromatin modifying enzymes and transcriptional machinery.

### C6 contributes to inhibition of ISRE-dependent gene expression during VACV infection

**Fig 9:**
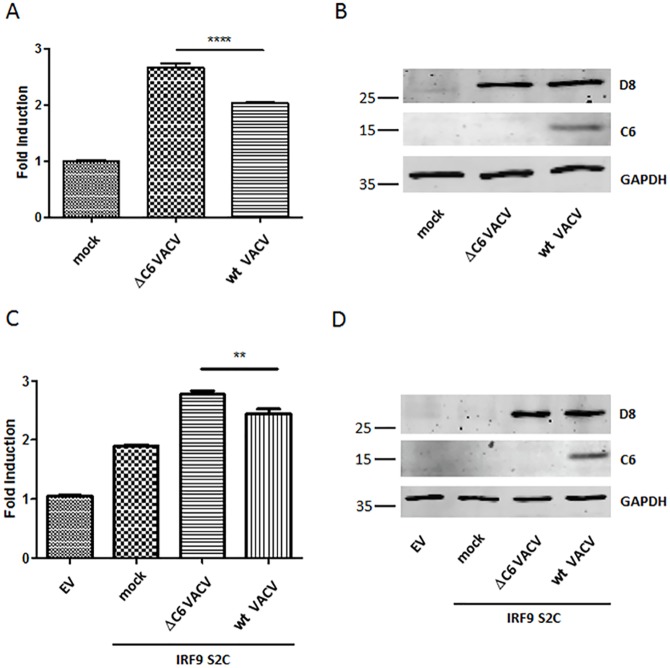
Protein C6 inhibits ISRE-dependent gene expression during VACV infection. (**A**) Multiple wells (n = 6 per condition) of a 96-well plate containing HEK293T cells were transfected with plasmids expressing firefly luciferase under the control of an ISRE promoter and a plasmid constitutively expressing renilla luciferase. Sixteen h post transfection, cells were either mock-infected or infected with vΔC6 or wt VACV at 5 PFU/cell and 5 h later the cells were harvested and the expression of firefly luciferase was measured and normalised to renilla luciferase expression. ****P<0.0001. (**B**) Lysates from cells treated as in (**A**) were subjected to SDS-PAGE and immunoblotting for VACV proteins D8 and C6, and for cellular GAPDH. (**C**) HEK293T cells were transfected by the same plasmids as described in (**A**) and 16 h later the cells were transfected with 100 ng/well (96-well plate) of a plasmid expressing IRF9-S2C for 4 h. The cells were then infected with vΔC6 or wt VACV at 5 PFU/cell for 5 h. The expression of firefly luciferase was measured and normalised to renilla luciferase expression. **P<0.01. (**D**) Lysates from cells treated as in (C) were analysed by SDS-PAGE and immunoblotting as in (**B**). In (**B**) and (**D**) the positions of molecular mass markers are shown in kDa on the left.

Finally, the biological importance of C6 for inhibition of the JAK-STAT pathway leading to activation of the ISRE promoter was assessed during VACV infection. The activity of C6 in blocking this pathway was likely to be masked to some degree during infection by the presence of the virus phosphatase VH1, which dephosphorylates STAT1 and STAT2 and is delivered into cells by the invading virion immediately after infection. Therefore, two methods were used to assess if C6 contributed to the inhibition of the JAK-STAT pathway during infection. One method was simply to transfect the ISRE-luciferase reporter plasmid into cells 16 h before the cells were infected with either wt VACV or the vΔC6 mutant and then measure ISRE-luciferase at different times p.i. Preliminary experiments established that infection of cells at 5 PFU/cell for 5 h was optimal before luciferase activity was measured in cell lysates. Under these conditions, infection by vΔC6 induced significantly greater luciferase activity than did wt VACV. Immunoblotting for VACV protein D8 showed that the virus infections were equivalent and immunoblotting for GAPDH showed equal loading of samples. This experiment was conducted using either crude or purified virus preparations (n = 4) and in each case a significant difference between the viruses was observed (Fig 9A).

The second method exploited the ability of the IRF9-S2C protein to activate ISRE promoter within the nucleus and downstream of the position at which the VH1 phosphatase mediates inhibition of the JAK-STAT pathway. Optimisation experiments to determine the amount of the IRF9-S2C plasmid to transfect and the length of time after transfection prior to virus infection showed that this potent inducer of ISRE-dependent gene expression was best only transfected a few hours before infection because overnight transfection induced very high levels of luciferase activity. Transfection of the IRF9-S2C plasmid for 4 h induced a modest 2-fold induction in luciferase activity and this was increased further by virus infection (Fig 9B). However, following virus infection for 5 h, wt VACV induced lower levels of luciferase than vΔC6, as in Fig 9A. These significant differences were seen reproducibly in multiple experiments (n = 4). Immunoblotting confirmed equal infection and protein loading.

Collectively, these data show that during VACV infection protein C6 is able to diminish expression from the ISRE promoter over and above the effect of the VH1 phosphatase.

## Discussion

Previously, C6 was identified as a VACV immunomodulator and virulence factor and was shown to inhibit the induction of type I IFNs through inhibition of the IRF-3/7 signalling pathway at the level of the TBK1/IKKε kinase complex [26]. This study identifies a second function for VACV protein C6 as an inhibitor of the cellular response to type I IFN. Data presented demonstrate that C6 inhibits IFNα-induced expression of ISGs at both the mRNA and protein level (Fig 1). The inability of a pharmacological inhibitor of TBK1/IKKε to reduce IFNα-induced reporter gene expression (S2 Fig) indicates that inhibition of this kinase complex by C6 is unlikely to explain the ability of C6 to also inhibit the cellular response to IFNα. Therefore, to elucidate the mechanism by which C6 has its inhibitory effect on this second pathway, the IFNα-induced phosphorylation and nuclear translocation of STAT1 and STAT2 were examined and C6 was found to have no effect on these early events of this signalling pathway (Figs 2, 4 and 5). Furthermore, both endogenous STAT2 and a constitutively active ISGF3 mimic, IRF-9-S2C, were still able to bind to the ISRE in the presence of C6 (Fig 7), indicating C6 exerts its inhibitory effect after ISGF3 binding to the ISRE. Interestingly, C6 interacts with STAT2 (Fig 8A–8C) and the transactivation domain (aa 747–851) of STAT2 fused to IRF-9 (Fig 8D) but not with STAT1 or IRF-9 (Fig 8A). The STAT2 transactivation domain is known to be required for the recruitment of chromatin modifiers and transcriptional machinery [37]. Therefore, the ability of C6 to interact specifically with this domain gives insight into how this viral protein may inhibit this crucial signalling pathway at such a late stage.

C6 is one of many VACV proteins that inhibit the IFN response, however, it is the first such protein known to inhibit both branches of the IFN response, inhibiting both IFNβ production and the cellular responses to type I IFN. C6 is also the first VACV protein identified to inhibit the response to type I IFN in the nucleus of infected cells; VACV inhibitors to date act early in the JAK/STAT signalling pathway, either extracellularly to prevent binding of secreted type I IFN to their receptor (B18) [13–15], or in the cytoplasm of infected cells to dephosphorylate activated STAT1 and STAT2 (VH1) [17,18]. C6 instead acts at a very late stage in the pathway, after the ISGF3 complex has formed, translocated into the nucleus and bound to the ISRE. Despite this, its inhibitory action was evident on 6 out of 7 of the ISGs examined, suggesting that the protein or step it targets is required for the induction of many ISGs.

The requirement for many protein inhibitors of a single, albeit important, signalling pathway is not well understood. However, VACV shows a similar ‘belt and braces’ approach to other signalling pathways, for example the NF-κB pathway, for which it is currently known to possess 10 inhibitors [9–12,23,29,30,38–42]. These proteins are not completely redundant however, as they cause virus attenuation *in vivo* when deleted individually [10,11,39,43–47]. Similarly, previous work has shown that deletion of C6 leads to an attenuated phenotype in both intradermal and intranasal models of VACV infection in mice [26], despite the presence of other IFN-signalling inhibitors. The identification of a second function of C6 means that the observed attenuation of the C6 deletion virus cannot be attributed to a single function as yet, but highlights the importance of this immunomodulatory protein. Structure-based mutagenesis of C6 may enable the dissection of these different activities as was done for the related VACV protein N1 [30].

The use of multiple proteins to inhibit a single pathway may be explained in many ways, such as the possibility of incomplete inhibition by any one protein, or the requirement for different immunomodulators in different cell types or infection stages. It could also be explained by possible crosstalk between innate immune signalling pathways meaning that inhibition of a pathway at a certain point could be overcome by activation of another communicating immune signalling pathway. However, the late stage at which C6 inhibits the type I IFN response would suggest that cross talk from other pathways would still be unable to activate ISGF3-driven ISG transcription in the presence of C6. The role of C6 in inhibiting the JAK-STAT pathway during virus infection may be masked to some degree by the effect of VH1 that dephosphorylates STAT1 and STAT2 rapidly after infection. VH1 is an essential gene for VACV replication [16], preventing its elimination by genetic manipulation. Nonetheless analysis of ISRE-driven gene expression early after infection with either wild type virus or a mutant virus lacking C6 showed a functional role for the C6 protein in diminishing ISRE-dependent gene expression (Fig 9). The roles of VH1 and C6 are therefore complementary. VH1 is expressed late in infection and packaged into the virion, whereas C6 is an early VACV protein, expressed from approximately 2 h post infection. These differential expression patterns might explain the requirement of both inhibitors, perhaps with their relative importance differing over the life cycle of the virus.

The principal transcriptional activating complex responsible for type I IFN-induced gene expression, ISGF3, consists of three components, STAT1, STAT2 and IRF-9. In this complex, STAT2 provides a potent and essential transcriptional activation domain [48]. The mechanistic process by which ISGF3, likely through this C-terminal domain of STAT2, signals to and promotes transcription of ISGs by RNA polymerase II remains unclear. However, a number of cellular proteins known to have roles in transcription, such as components of the Mediator complex [49], or chromatin modification, including histone deacetylases (HDACs) [50], histone acetyltransferases (HATs) [51] and chromatin remodeling complexes [52–55], have been identified as being essential for ISGF3-driven transcription. The precise mechanism by which C6 inhibits JAK/STAT signalling remains to be determined, however it is possible that the association of C6 with the STAT2-transactivation domain could prevent or alter the interactions of STAT2 with these or other cellular proteins required for ISG transcriptional induction. Indeed some such proteins have been shown to bind directly to the STAT2 transactivation domain, for example the HATs p300/CBP [51] and GCN5 [56]. In the future, further work is needed to assess the exact consequence of the C6-STAT2 TAD interaction.

The importance of the IFN-signalling pathway in preventing viral replication and spread dictates that the majority of mammalian viruses have one or multiple mechanisms of inhibiting the response of infected cells to type I IFN. There is an array of different mechanisms by which viral proteins inhibit JAK/STAT signalling, some of which are reviewed in [4]. Many such mechanisms focus on inhibiting the early steps in the JAK/STAT signalling pathway, by degradation of either STAT1 or STAT2 as with PiV5 [32] and PiV2 V proteins [57], respiratory syncytial virus NS1 and NS2 proteins [58] and dengue NS5 protein [59], by inhibition of STAT phosphorylation as with Sendai virus C protein [60], or by cytoplasmic sequestration of the ISGF3 complex as with NiV [31] and Hendra virus [61] V proteins.

Fewer viral proteins have been identified that inhibit the later stages of this signalling pathway, once the ISGF3 complex has reached the nucleus. The human cytomegalovirus (HCMV) IE1 protein interacts with STAT2 and inhibits the binding of STAT2 and promyelocytic leukemia protein (PML), a protein that associates with STAT1, STAT2, and HDACs, to ISG promoters [62]. Therefore, HCMV IE1 delivers its inhibitory action within the nucleus and via an interaction with STAT2, but again upstream of the inhibitory action of C6. Conversely, adenovirus E1A protein has its inhibitory effect, like C6, downstream of ISGF3- promoter binding but does so through interacting with and preventing the functioning of HATs and histone ubiquitylating complexes required for full ISG transcriptional activation and not through a direct interaction with STAT2 [63]. Similarly, influenza A virus nonstructural protein 1 (NS1) inhibits the cellular response to type I IFNs in the nucleus through interaction with a complex involved in transcriptional elongation, hPAF1C and once again not by a direct interaction with STAT2 [64]. Interestingly, NS1 inhibits type I IFN production through binding dsRNA produced by the virus and thus preventing its detection by PRRs [65–67]. NS1 and C6 have therefore both evolved to inhibit both type I IFN production and type I IFN-induced signalling but by distinct mechanisms in each pathway.

To our knowledge VACV protein C6 is the first viral protein shown to both associate with the STAT2 transactivation domain and inhibit IFNα-dependent ISG induction after ISGF3 binding to the ISRE. The sequence of events that occur following ISGF3 binding to the ISRE is poorly understood and further elucidating the mechanism by which C6 inhibits this signalling pathway in the nucleus may enhance our knowledge of the late stages of the type I IFN signalling pathway. Lastly, it is notable that although VACV is a cytoplasmic DNA virus, it has evolved mechanisms to inhibit IFN production or activity within both the cytoplasm and the nucleus and indeed outside the infected cell by the expression of soluble type I and type II IFN binding proteins.

## Materials and Methods

### Cell lines and viruses

HEK293T (ATCC CRL-11268) cells were maintained in Dulbecco’s Modified Eagle’s Medium (DMEM, Invitrogen) with 10% heat treated (56°C, 1 h) foetal bovine serum (FBS, Seralab) and penicillin/streptomycin (P/S, 50 μg/ml, PAA laboratories). HeLa (ATCC CCL-2) cells were maintained in Minimum Essential Medium (MEM, Invitrogen) supplemented with 10% FBS, P/S and 1:100 non-essential amino acids (Gibco).

HeLa cells stably expressing GFP only or in combination with V5-tagged C6 (V5-C6), V5-Parainfluenza virus 5 V protein (V5-PiV5-V) or V5-tagged B14 (V5-B14), were obtained after transduction of cells with lentiviruses (see below) and sorting to obtain GFP-positive cells in a MoFlo MLS high-speed cell sorter (Beckman Coulter). For each protein two populations were sorted based on GFP expression level; the top 30% of GFP-expressing cells (high expressers) and the next 30% (middle expressers). Immunoblot analysis was used to determine the expression levels of the protein of interest in these two populations. Based on the observed expression of these proteins, the high GFP-expressing populations were chosen for, EV (GFP only), V5-C6, and V5-PiV5-V and the middle GFP-expressing V5-B14 population. For protein expression level of cell lines see S1C Fig. Lentivirus particles for transduction were generated after transient co-transfection of HEK293T cells with entry and packaging vectors and the bicistronic genomic vector encoding GFP and the appropriate V5 tagged protein using PEI (CellnTec).

The recombinant VACV Western Reserve strain C6-TAP and N1-TAP viruses were described [68]. The tandem-affinity purification (TAP) tag used here and in plasmids described below contains 2 copies of the streptavidin-binding sequence and 1 copy of the FLAG epitope [69]. Wild type (wt) VACV strain WR and the deletion mutant lacking the *C6L* gene were described [26].

### Antibodies and reagents

Antibodies used were from the following sources; Rabbit (Rb) anti-FLAG (Sigma-Aldrich, F7425, diluted 1:5000), Mouse (Ms) anti-V5 (AbD Serotec Ltd, MCA1360, diluted 1:5000), Rb anti-HA (Sigma Aldrich, H6908, diluted 1:1000), Ms anti-α-tubulin (Millipore, 05–829, diluted 1:5000), Rb anti-actin (Sigma, A2066, diluted 1:1000), Ms anti-Phospho-STAT1 (Invitrogen, 333400, diluted 1:750), Rb anti-phospho-STAT2 (Millipore, 07–224, diluted 1:1000), Rb anti-STAT1 for immunofluorescence (Millipore, 06–501, diluted 1:300), Rb anti-STAT1 for western blotting (Cell signalling, 9172S, diluted 1:1000), Rb anti-STAT1 for immunoprecipitation (Santa Cruz, sc-345, diluted 1:100), Rb anti-STAT2 (Santa Cruz, sc-476, diluted 1:100 for immunofluorescence and 1:500 for western blotting), Ms anti-Ku70 (Abcam, ab3114, diluted 1:1000), Ms anti-IFIT1 for flow cytometry (Abcam, ab70023, 1:500), and Alexa Fluor 546 goat anti-Rb IgG (H+L) (Invitrogen, A-11010, diluted 1:750 for immunoblotting) or Alexa Fluor 647 donkey anti-Ms IgG (H+L) (Invivogen, A- 31571, diluted 1:2000 for flow cytometry).

Reagents used in this study were BX795 (Tocris), poly(I:C) (InvivoGen), Protein G Sepharose 4 Fast Flow (GE Healthcare), High Capacity Streptavidin Agarose Resin (Thermo Scientific), human IFNα and human IL-1β were from Peprotech, poly(dI:dC) and ANTI-FLAG M2 Affinity Gel were from Sigma Aldrich. Biotinylated DNA for immunoprecipitations were synthesised by Integrated DNA technologies. The sequences were ISREcore; TGCCTCGGGAAACCGAAACTGAAGCCA and ISRErandom ACTGATCGGAAACCGAAACGATCTATG. These sequences were taken from [36].

### Plasmids

Codon-optimised TAP-C6 and N1-TAP, were described previously [68] and [30]. B14-TAP was kindly provided by Dr. Brian Ferguson (Department of Pathology, University of Cambridge, UK). The sequence of PiV5-V and NiV-V were amplified by PCR from plasmids kindly provided by Prof. Richard Randall (University of St Andrews, UK) and then subcloned into mammalian expression vectors pcDNA3.1 (Invitrogen) with an N-terminal V5 tag. V5-C6 was produced by PCR amplification of C6 from VACV WR DNA and cloned into pcDNA3.1. GFP-V5 was provided by Dr. Christian Ku (Department of Pathology, University of Cambridge). The sequences of IRF-9, STAT1 and STAT2 were amplified by PCR from HeLa cDNA and subcloned into mammalian expression vector pcDNA4/TO with a C-terminal TAP tag and/or vector pcDNA3.1 with a N-terminal HA tag. pcDNA3 IRF9-STAT2C was a gift from Prof. Curt Horvath (Addgene plasmid 37544) [35]. pcDNA4/TO (Invitrogen) was used in luciferase reporter assays as EV. ISRE-luciferase, NF-κB-Luciferase, and TK renilla were obtained from Dr. Andrew Bowie (Trinity College, Dublin, Ireland), and ISG56.1-Luciferase was from Ganeth Sen (Lerner Research Institute, Ohio, USA).

### Reporter gene assays

Reporter gene assays were performed in HeLa or HEK293T cells seeded in 96-well plates. Cells were transfected with 100 ng firefly luciferase reporter plasmid, 10 ng GL3-renilla luciferase plasmid and 100 ng of expression plasmid for the protein of interest. For the BX795 reporter gene assay only the firefly report plasmid and GL3-Renilla plasmids were transfected. For the IRF9-S2C reporter gene assays 100 ng firefly reporter plasmid and 10 ng GL3-Renilla plasmid were transfected along with 50 ng IRF9-S2C and 50 ng C6 expression vector or control plasmids, except in the empty vector only control where 100 ng pcDNA4 was transfected only. Transit-LT1 (Mirus, 2 μl per 1 μg DNA) was used for transfection of HeLa cells and PEI (CellnTec, 2 μl per 1 μg DNA) for HEK-293T cells. Sixteen hours post transfection cells were stimulated as indicated in the figure legends. Cells were harvested in passive lysis buffer (Promega, 100 μl/well). The firefly-luciferase readings of each sample were normalised to the renilla-luciferase readings and fold inductions were calculated relative to the non-stimulated controls for each plasmid. Experiments were performed in triplicate and conducted at least 3 times.

### Real time PCR

HeLa cell lines stably expressing the proteins of interest were grown in 12-well plates and RNA was extracted using the RNeasy kit (QIAGEN). One μg of each RNA sample was used to synthesise cDNA using Superscript III reverse transcriptase according to the manufacturer’s protocol (Invitrogen). ISG mRNA was quantified by real-time PCR using a ViiA 7 Real-Time PCR System (Life Technologies), fast SYBR Green Master Mix (Applied Biosystems) and the following primers, IFIT1 (Fwd: CCTGAAAGGCCAGAATGA GG, Rev: TCCACCTTGTCCAGGTAAGT) IFIT3 (Fwd: ACACAGAGGGCAGTCATGAGTG, Rev: TGAATAAGTTCCAGGTGAAATGGC) MxA (Fwd: ATCCTGGGATTT TGGGGCTT, Rev: CCGCTTGTCGCTGGTGTCG) GAPDH Fwd: ACCCAGAAGACTGTGGATGG, Rev: TTCTAGACGGCAGGTCAGGT). Amplification of ISGs was normalised to glyceraldehyde-3-phosphate dehydrogenase (GAPDH) amplification from the same sample, and the fold induction of genes in response to IFNα was calculated relative to the unstimulated control of the cell line. Experiments were performed in biological triplicate and conducted three times.

### Flow cytometry

HeLa cells stably expressing the proteins of interest were grown in 6-cm dishes and stimulated with 500 U/ml IFNα or mock-stimulated for 8 h. Cells were removed from the dishes by addition of trypsin (GIBCO), transferred to Eppendorf tubes and washed twice with ice-cold PBS. Cells were fixed in 4% paraformaldehyde and permeabilised with 0.1% Triton X. Cells were then incubated with anti-IFIT1 (Abcam, ab70023) in Triton buffer (0.5% BSA, 0.02% sodium azide, 0.1% Triton X-100 in PBS) for 1 h at 4°C. Cells were washed twice with Triton buffer and then incubated with Alexa 647 Donkey anti-Mouse (Invivogen) in Triton buffer for 1 h in the dark at room temperature. Cells were washed twice in Triton buffer, once in 0.5% BSA in PBS and then analysed on a CyAn ADP Analyser (Beckman coulter). Collected data were analysed using Summit (Beckman Coulter).

### Confocal microscopy

HeLa cells stably expressing the proteins of interest were grown on glass coverslips in 6-well plates. Cells were stimulated with 1000 U/ml IFNα for 1 h. Cells were fixed with 4% paraformaldehyde. Auto-fluorescence was quenched in 150 mM ammonium chloride in PBS and the cells were then permeabilised in 0.1% Triton X-100 in PBS. Cells were incubated in blocking buffer (0.5% BSA in PBS) for 30 min, stained with primary antibody for 1 h (STAT1: 1:300, STAT2 1:100 in blocking buffer) and for 1 h with secondary antibody (Alexa 546, Invitrogen). Coverslips were mounted onto microscope slides in Mowiol 4–88 containing 4',6-diamidino-2-phenylindole (DAPI). Slides were visualised and imaged using a Zeiss LSM 780 Confocal microscope. Images were viewed using LSM Image Browser (Zeiss).

### Co-immunoprecipitation

HEK293T cells were grown in 10-cm dishes and transfected with the constructs outlined in the figure legends using either Transit LT1 (Mirus) or calcium phosphate transfection. Sixteen hours later cells were stimulated with IFNα or mock-treated as described in figure legends, then lysed in lysis buffer (150 mM NaCl, 20 mM Tris-HCl pH 7.4, 10 mM CaCl_2_, 0.1% (v/v) Triton-X, 10% (v/v) glycerol and protease (cOmplete Mini, Roche) and phosphatase inhibitors (PhosSTOP, Roche)) and cleared by centrifugation. Samples were then incubated with 30 μl Protein G Sepharose 4 Fast Flow (GE Healthcare) and anti-STAT1 (Santa Cruz, sc-345) for 6 h, or ANTI-FLAG M2 Affinity Gel (Sigma Aldrich) or Anti-HA Agarose (Sigma Aldrich) and 2 h. Immunoprecipitations were washed 3 times in lysis buffer and bound proteins were eluted by boiling in buffer containing SDS. Samples were then analysed by SDS-PAGE (polyacrylamide gel electrophoresis) and immunoblotting with the stated antibodies.

### Biotinylated-DNA immunoprecipitation

HEK293T cells were grown in 10-cm dishes and co-transfected with expression plasmids for IRF9-S2C, and V5-NiV-V, V5-C6 or V5-GFP using calcium phosphate transfection in triplicate for each condition. Sixteen hours later cells were lysed in lysis buffer (25 mM Tris-HCl pH 7.4, 150 mM NaCl, 1 mM EDTA, 1% NP40, 5% glycerol and protease (cOmplete Mini, Roche) and phosphatase (PhosSTOP, Roche) inhibitors). Lysates were incubated firstly with 10 ng/ml poly(dI:dC) for 30 min, then with 100 pmol biotin-labelled ISREcore or biotin-labelled control DNA for 1.5 h and finally with 30 μl High Capacity Streptavidin Agarose Resin (Thermo Scientific) for 3.5 h. Immunoprecipitations were washed four times with lysis buffer and proteins were eluted by boiling in buffer containing SDS. Samples were then analysed by SDS-PAGE and immunoblotting with the stated antibodies.

### Statistical analysis

Un-paired student’s T-tests were used to analyse data, with Welch’s correction applied when variances differed significantly between samples. Statistical significance is expressed as: *P<0.05, **P<0.01, ***P<0.001 and ****P<0.0001.

## Supporting Information

S1 Fig Expression levels of C6 and control proteins in reporter gene assays and stable cell lines. Immunoblot analysis of protein expression from transiently transfected HEK293T (**A**) and HeLa (**B**) cells in a 96-well plate format for dual luciferase assays shown in Fig 1A and 1B, respectively. (**C**) Expression level of V5-tagged proteins from stably transduced HeLa cell lines used in Figs 1C, 1D, 2 and 4. Immunoblots were performed at least twice and a representative figure is shown. Positions of molecular mass markers are shown to the left of the immunoblots.
(TIF)

S2 Fig Inhibition of TBK1/IKKε does not inhibit IFNα- induced reporter gene expression. HeLa cells were transfected with plasmids expressing firefly luciferase under the control of the stated promoters and constitutively expressing renilla luciferase. Sixteen hours post transfection cells were treated with BX795 (0.5 μM) or DMSO for 3 h, after which they were treated with pI:C (20 ug/ml), IL-1β (50 ng/ml), or IFNα (250 U/ml) as appropriate. Results are shown as percentage induction of reporter gene relative to the firefly luciferase induction following appropriate stimulation in DMSO-treated control cells. P<0.001.
(TIF)
