# Supplementary figures and images for: Vaccinia Virus Protein C6 Inhibits Type I IFN Signalling in the Nucleus and Binds to the Transactivation Domain of STAT2
Source: PLoS Pathog. 2016 Dec 1;12(12):e1005955. doi: 10.1371/journal.ppat.1005955 (PMC5131898; doi:10.1371/journal.ppat.1005955)

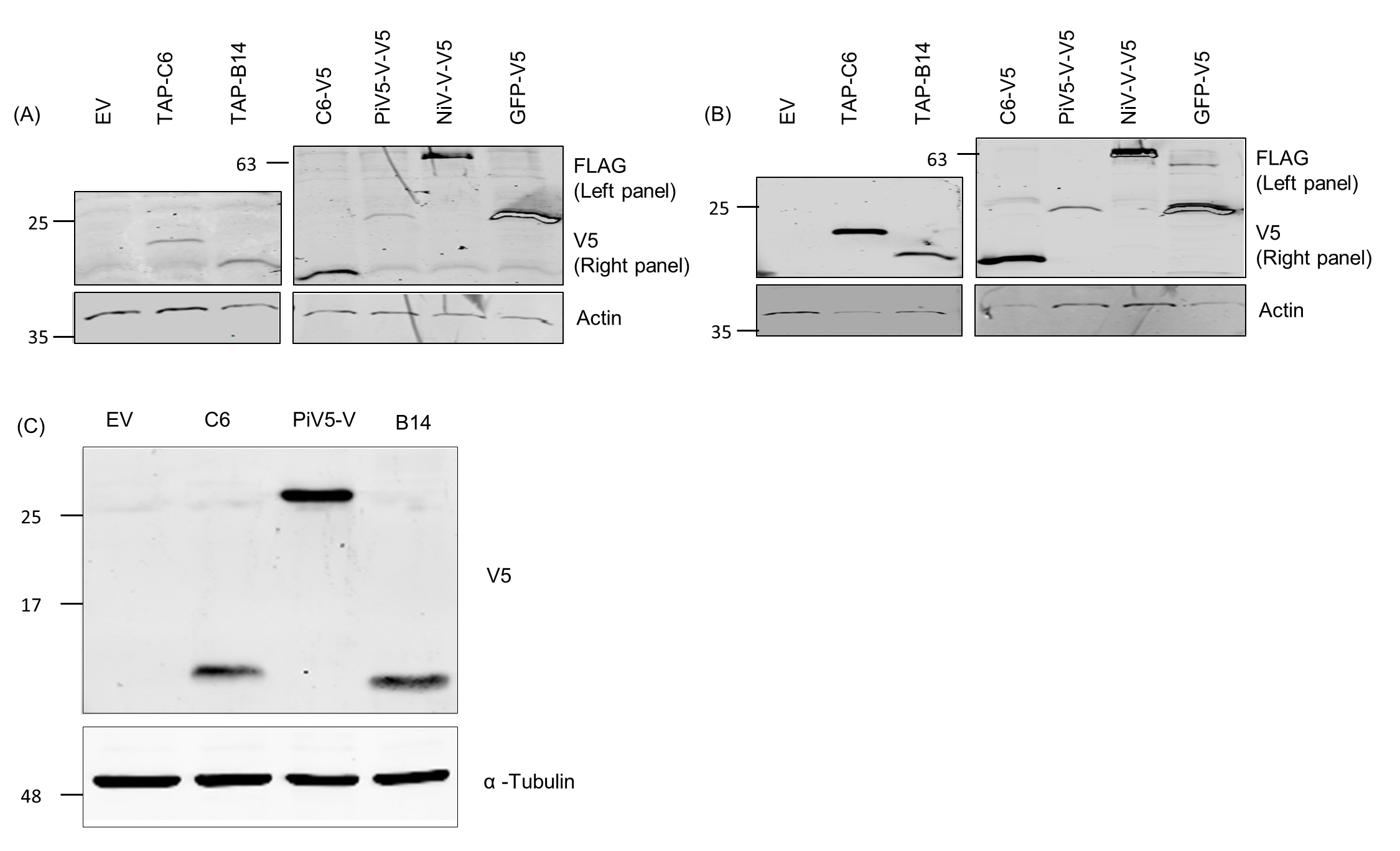

Supplement: S1 Fig — Immunoblot analysis of protein expression from transiently transfected HEK293T (A) and HeLa (B) cells in a 96-well plate format for dual luciferase assays shown in Fig 1A and 1B, respectively. (C) Expression level of V5-tagged proteins from stably transduced HeLa cell lines used in Figs 1C, 1D, 2 and 4. Immunoblots were performed at least twice and a representative figure is shown. Positions of molecular mass markers are shown to the left of the immunoblots. (TIF) [file ppat.1005955.s001.tif]

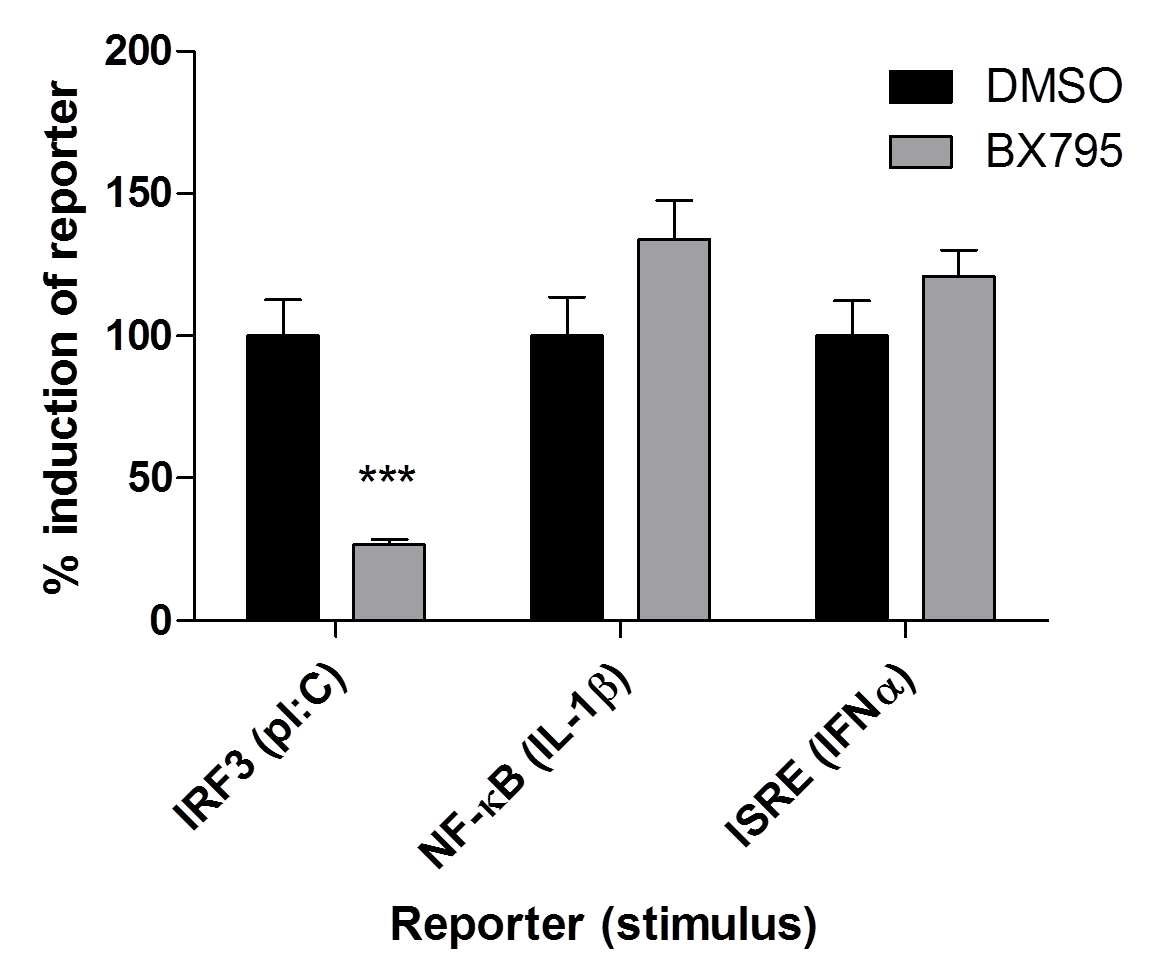

Supplement: S2 Fig — HeLa cells were transfected with plasmids expressing firefly luciferase under the control of the stated promoters and constitutively expressing renilla luciferase. Sixteen hours post transfection cells were treated with BX795 (0.5 μM) or DMSO for 3 h, after which they were treated with pI:C (20 ug/ml), IL-1β (50 ng/ml), or IFNα (250 U/ml) as appropriate. Results are shown as percentage induction of reporter gene relative to the firefly luciferase induction following appropriate stimulation in DMSO-treated control cells. P<0.001. (TIF) [file ppat.1005955.s002.tif]
